# Impact of Gamification on Consumers’ Favorability in Cause-Related Marketing Programs: Between-Subjects Experiments

**DOI:** 10.2196/35756

**Published:** 2023-01-10

**Authors:** Yanhe Li, Yanchen Li, Xiu Zhou, Kunshu Ma

**Affiliations:** 1 School of Management Zhejiang Shuren University Hangzhou China; 2 School of Business Macau University of Science and Technology Macau S.A.R China; 3 School of Accounting and Finance Beijing Institute of Technology Zhuhai China; 4 School of Management Southwest Minzu University Chengdu China

**Keywords:** cause-related marketing, gamification, enjoyment, favorability, business, marketing, gamified

## Abstract

**Background:**

Successful cause-related marketing (CRM) campaigns can help companies stand out from their competitors; however, CRM may not have pleasant outcomes, even if it receives substantial investment.

**Objective:**

This research aimed to investigate how gamified CRM projects influence consumers’ favorability.

**Methods:**

We introduced 3 different CRM projects in 3 different studies. Every project had 2 versions according to the level of gamification, and participants were randomly assigned into these 2 groups. Additionally, we used a 2 (gamification: lower, higher) 2 (rules presentation: without visual cues, with visual cues) between-subjects design to test the moderation role of rules presentation in gamified CRM projects.

**Results:**

In Study 1, we identified that the highly gamified CRM program induces more enjoyment (*F*_1,139_=21.11, *P<*.001) and higher favorability (*F*_1,139_=14.57, *P<*.001). Moreover, we found that enjoyment played a mediation role between gamification and favorability (*P<*.001) in Study 2. In addition, the results of Study 3 indicated rules presentation in a gamified CRM program can moderate the indirect effect of gamification on favorability via enjoyment (index of the moderated mediation: 95% CI –1.12 to –0.10; for rules presentation with visual cues: 95% CI 0.69 to 1.40; for rules presentation without visual cues: 95% CI 0.08 to 0.83).

**Conclusions:**

Overall, this research contributes to the CRM literature and suggests gamification is an effective way of managing CRM campaigns.

## Introduction

### Background

Corporate social responsibility practices are currently popular for firms to enable themselves to stand out from their competitors and achieve sustainable performance [1]. Generally, companies undertake cause-related marketing (CRM) as one solution to show their commitment to corporate social responsibility [2]. Successful CRM campaigns can help companies elicit positive attitudes [3] and emotional bonds between them and their customers [4]. However, CRM may not have pleasant outcomes, even if it receives substantial investment [5,6]. It is therefore critical to understand the related predictors so that practitioners can develop CRM activities that will have desirable outcomes. 

Gamification is a new and effective way to generate CRM success. Recently, some companies have applied gamification to their CRM practices. For example, Alipay, one of the famous e-payment applications in China, launched a gamified CRM scheme called “Ant Forest” in 2016. Users grow virtual trees in Ant Forest by collecting game points. As users complete the game tasks, they earn points for taking buses, walking daily steps, or using online payment in a low-carbon lifestyle. After obtaining enough points, users can turn their virtual trees into real ones and thus contribute to environmental protection. The hedonic benefit provided by a gamified system motivates users to take action. However, to our best knowledge, few studies discuss gamified CRM and its impact on people's attitudes or behaviors [7]. In this article, we advanced previous research and performed empirical studies to answer related questions.

### Enjoyment and Favorability

Consumers are not totally rational. As they make purchase decisions, they involve their fantasies, emotions, or other experiential perspectives rather than just processing the information [8].

Gamification is thus the popular choice for practitioners to satisfy consumers with utilitarian values, hedonic values, and social values [9]. On one hand, the concept of gamification originates from “game.” Gamification indicates the use of game design elements, game mechanisms, or games in nongame contexts [10]. It aims to provide consumers with positive emotional and involved experiences (ie, gameful experiences) [11]. On the other hand, some research (eg, Jung et al [12], Kim and Ahn [13], and Sailer et al [14]) has proved that game design elements (eg, points, badges, and leaderboards) can not only evoke users' intrinsic motivations and yield performance gains but also internalize extrinsic motivations (eg, external rewards).

﻿Therefore, we believed that, compared with traditional CRM activities, consumers enjoy more hedonic values and feel as if they are more involved in gamified activities, leading to the following hypothesis: (H1) Gamification has a positive influence on enjoyment.

Consumers do not adopt CRM activities casually [6]. Consumers’ personal evaluation of or beliefs around the cause can influence the success of CRM [15]. Thus, in this study, we defined favorability as consumers' positive attitudes toward CRM activities [16,17], to measure the success of CRM programs.

Generally, CRM campaigns work when consumers benefit from feeling pleasure during participation [18]. We posited that consumers are more willing to accept gamified CRM activities, since they can offer more opportunities for consumers to interact with the cause and the perceived enjoyment from gamified CRM campaigns positively influence consumers’ favorability.

Extant research suggests that positive emotions can arouse individuals' positive attitudes [19]. Especially in the context of CRM, hedonic products that are associated with positive emotional processing are more likely to provoke emotional contagion; individuals thus have more favorable attitudes toward the CRM programs [20].

Although studies examining the relationship between enjoyment and favorability in the context of gamified CRM are lacking, various studies (eg, Yang et al [21], Catalán et al [22], and Hwang and Choi [23]) validated the positive influence of perceived enjoyment from gamification on positive attitudes, such as consumers’ brand attitudes [24], continuous use intention [25], and brand loyalty [26].

Therefore, we hypothesized that (H2) enjoyment has a positive influence on favorability and mediates the relationship between gamification and favorability.

### Rules Presentation

Gamification embraces the nature of games. Importantly, rules are the core factors of games [27]. Containing various game mechanics [28], rules stipulate how to achieve the goal of a game and the outcome of each trial [29].

In this article, we defined rules presentation as the way of presenting the rules of a CRM project, with or without visual cues. We believe that the way rules are presented can influence people's enjoyment. Rules with visual cues tend to help people to process information fluently and thus induce enjoyment at a higher level. According to processing fluency theory, the ways of processing information vary from the degrees of effort and speed [30]. With a fluent process, it is faster and easier to make judgments; however, a process that is not fluent has the opposite effect.

Generally, individuals prefer a stimulus that is easier to perceive and tend to favor it [31-33]. Thus, fluency always results in positive outcomes [34]. Past research has shed light on that fact that fluency of information processing (eg, rules processing) leads to consumers' positive affective responses (eg, Janiszewski [35] and Gamblin et al [36]) or leads to purchase intentions (eg, Zhang et al [37], Jaud and Melnyk [38], and Wang et al [39]).

Meanwhile, fluent processes are always involved in visual categorization [40,41]. When faced with experiential-attribute (ie, affective or sensory) targets, consumers are more likely to receive stimuli and respond immediately, without elaborating or reasoning [42].

Therefore, we hypothesized that, if the rules of gamified projects are performed with visual cues (eg, in figurative or other sensory ways), consumers tend to perceive higher enjoyment. On the contrary, however, when the rules are presented in plain and monotonous literal words, consumers perceive less or even no enjoyment from gamified CRM programs. We thus predicted that (H3) visual cues in rules presentation moderate the relationship between gamification and enjoyment. People tend to have a higher level of enjoyment under the condition with visual cues (vs the condition without visual cues).

**Figure 1:**
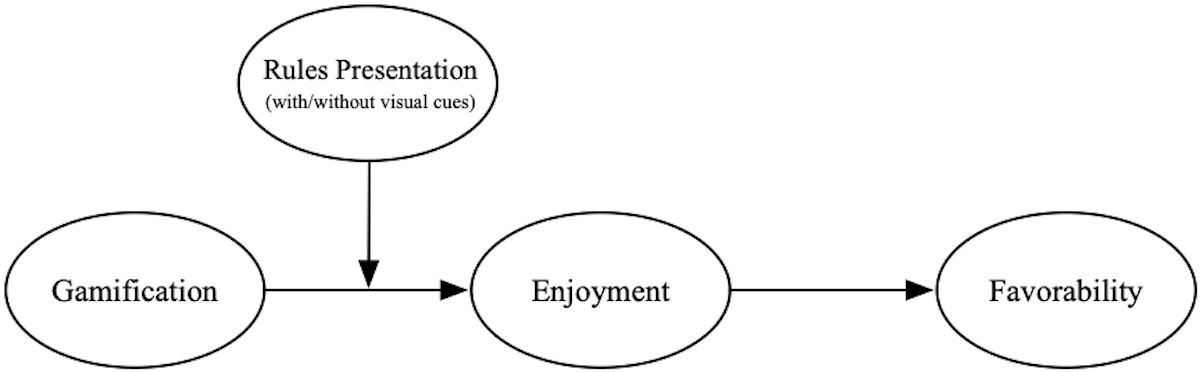
Conceptual model.

The conceptual model is illustrated in Figure 1.

Overall, this research aimed to test if consumers have a higher level of favorability to respond in the context of gamification and try to explain why gamification strategy can work. Specifically, we hypothesized that gamification has a positive influence on enjoyment and consumers’ favorability toward gamified CRM programs and that enjoyment mediates the relationship between gamification and favorability. Moreover, we also hypothesized that visual cues in rules presentation can moderate the relationship between gamification and enjoyment. People tend to have a higher level of enjoyment under the condition with visual cues (vs the condition without visual cues). We described 3 studies in detail to test our hypotheses. Finally, we addressed the theoretical and managerial implications of this research and put forward some future research directions as suggestions.

## Methods

### Study Design in Study 1

We created 2 videos to introduce the Ant Forest of Alipay. The higher gamification version illustrated the real-life version of Ant Forest. For example, users can gain points by finishing certain assignments or games or stealing from friends. After accumulating enough points, they can turn their virtual plants into real ones. However, the lower gamification version presented a fictitious Ant Forest interface. We deleted all the add-in games. Specifically, users could only accumulate points from conducting eco-friendly behaviors (eg, using public transit) but could not accumulate points from other highly gamified methods (eg, finishing games or interactions with friends). Since the basic rules were retained, we regarded the fictitious version as a lower gamified program. To minimize noise, we kept 2 versions of the same visual designs (eg, images) and presentation length (ie, 30 seconds).

A pretest was first conducted to show that the 2 versions of Ant Forest were not significantly different in relevant factors except for the level of gamification. In the pretest, we told the participants to evaluate the given Ant Forest programs. Participants were randomly assigned to 1 of 2 conditions. We performed a confounding check of brand trust, brand attitude, and brand familiarity (7-point scales; see Multimedia Appendix 1 for details [43-45]). We then asked participants to rate the perceived gamification level. The measurement was adapted from that of Hwang and Choi [23]: “This Ant Forest program entails a game component.” (1=Strongly Disagree; 7=Strongly Agree).

After the pretest, we collected a new data sample to conduct our main study. In the main study, we first provided 2 versions of the Ant Forest introduction videos and asked participants to rate their willingness to favor the programs. The measurement was adapted from that of Speed and Thompson [46]: “This form of Ant Forest makes me feel more favorable toward the program.” “This form of Ant Forest would improve my perception of the program.” “This form of Ant Forest would make me like the program more.” (1=Strongly Disagree; 7=Strongly Agree; =.78). Second, to measure enjoyment, we used the scale from Höllig et al [47]: “I would find the program presented in the video game enjoyable.” “I would find the program presented in the video game enjoyable and pleasant.” “I would find the program presented in the video game enjoyable and exciting.” “I would find the program presented in the video game enjoyable and interesting” (1=Strongly Disagree; 7=Strongly Agree; =.81). Third, we asked the participants to rate the perceived gamification level for our manipulation check. Finally, we also performed a confounding check.

### Study Design in Study 2

In Study 2, we focused on the relationships among gamification, enjoyment, and favorability and tested if enjoyment plays the role of mediator (H2). We proposed that individuals are more likely to perceive higher enjoyment from a gamified CRM program and in turn have higher favorability toward the program (H2). In Study 2, we created a new stimulus to test the hypothesis. Moreover, to provide generalizability for our results related to H1, we also tested them in this study.

Similar to Study 1, we created 2 videos to introduce a CRM program sponsored by Douyin. Douyin (also known as “TikTok” in western countries) is a popular video-sharing social network service in China. The program is named “Dream Fairy” and aims to donate books to children. The higher gamification version was the real-life version of Dream Fairy in which users can gain points by watching or publishing instant videos, participating in question-and-answer games, or interacting with friends. When they have accumulated enough points, they can make their donations in reality. On the contrary, the lower gamification version presented a fictitious Dream Fairy interface. We deleted all the games. Specifically, users could only accumulate points from watching instant videos or lives and from publishing instant videos. They could not accumulate points from other highly gamified methods (eg, playing games or social interactions). We also kept 2 versions of the same image designs and presentation duration (ie, 30 seconds) to minimize noise in the data. In Study 2, we also performed a pretest and a main study to verify the internal validity.

### Study Design in Study 3

In Study 3, we conducted the mediation analysis again as a robustness check and explored the moderation role of visual cues between gamification and favorability.

We proposed that rules presentation moderates the effect of gamification on enjoyment and finally influences individuals’ favorability toward CRM (H3). In Study 3, we provided the rules to participate in a CRM either with or without visual cues. Presumably, people tend to process rules with visual cues more fluently and thus perceive more enjoyment. Therefore, we anticipated that, when rules are presented with visual cues, a higher (vs lower) gamified CRM facilitates higher favorability.

In Study 3, we chose a CRM program called “Protect Pandas” as the stimuli material. This CRM program aims to protect pandas and is sponsored by Weibo, a quite trendy mobile application in China.

Similar to previous studies, we created 2 videos to introduce the CRM program. The higher gamification version was the true version in reality. For example, users can gain points by logging into the app; publishing, reading, sharing, or liking posts; commenting; or interacting with friends. After accumulating enough points, Weibo helps users to plant actual bamboo to feed pandas. On the contrary, the lower gamification version presented a fictitious interface. The higher game elements were all deleted. Users could not accumulate points from social interactions with friends but only from daily routine behaviors in the app (eg, publishing or reading posts). We kept 2 versions in the same image designs and presentation duration (ie, 30 seconds) to avoid noise in the data. A pretest and a main study were also conducted in Study 3.

### Ethical Approval

The university institutional review board at Zhejiang Shuren University approved all data collection procedures and scales we used (20221225).

### Data Source

All the data in this research were collected in Credamo, which is an online survey platform. The questionnaires were randomly distributed to a registered consumer panel by the platform system, and researchers can collect data in exchange for monetary compensation. All the studies performed in this research were in accordance with the Checklist for Reporting Results of Internet E-Surveys (CHERRIES). All participants provided written consent to participate in the research and were informed that they had the ability to opt out.

## Results

### Results of Study 1

#### Pretest

We recruited 50 participants from Credamo. The results revealed that there were no significant differences between the 2 conditions for brand trust (lower gamification mean 6.19; higher gamification mean 6.42; *P*=.17), brand attitude (lower gamification mean 6.04; higher gamification mean 6.22; *P*=.38), or brand familiarity (lower gamification mean 6.12; higher gamification mean 6.35; *P*=.41). As expected, levels of perceived gamification were significantly different between the 2 conditions (lower gamification mean 3.87; higher gamification mean 5.91; *F*_1,48_=31.63, *P<*.001).

#### Main Study

We recruited 141 participants from Credamo; 78 (55.3%) were female, and 129 (91.5%) were aged from 21 years to 40 years. All the confounding variables (eg, brand trust, brand attitude, and brand familiarity) showed no significant differences (*P*=.09, *P*=.07, *P*=.19, respectively). Since all the participants were randomly separated into groups, we did not discuss any covariates (eg, age) further. We also performed the manipulation check on the variable of perceived gamification. The result revealed that the levels of perceived gamification were different between the 2 groups (lower gamification mean 4.89; higher gamification mean 5.83; *F*_1,139_=32.63, *P*<.001).

We then took enjoyment as a dependent variable and conducted a similar analysis. The result showed that gamification has a positive influence on enjoyment (lower gamification mean 5.44; higher gamification mean 6.04; *F*_1,139_=21.11, *P<*.001; partial ƞ^2^=0.13), which means the higher gamified program leads to higher enjoyment than the lower gamified program.

We finally took favorability as the dependent variable and conducted a similar analysis. The result showed that gamification has a positive influence on favorability (lower gamification mean 5.63; higher gamification mean 6.13; *F*_1,139_=14.57, *P<*.001; partial ƞ^2^=0.09), which means the higher gamified program leads to higher favorability than the lower gamified program.

﻿The results of Study 1 supported H1. The highly gamified CRM program induces more enjoyment and higher favorability.

### Results of Study 2

#### Pretest

A pretest was conducted with 42 participants recruited from Credamo. Participants were randomly assigned to 1 of 2 conditions and were instructed to evaluate the given programs. We also performed a confounding check of brand trust, brand attitude, and brand familiarity. The results revealed that there were no significant differences between the 2 conditions for brand trust (lower gamification mean 5.66; higher gamification mean 5.66; *P*=.98), brand attitude (lower gamification mean 6.11; higher gamification mean 5.91; *P*=.40), or brand familiarity (lower gamification mean 6.05; higher gamification mean 6.13; *P*=.79). However, perceived gamification differed between the 2 conditions (lower gamification mean 4.21; higher gamification mean 5.83; *F*_1,40_=10.81, *P*=.002).

#### Main Study

We recruited 114 participants from Credamo; 55 (48.2%) were female, and all the participants were aged from 21 years to 40 years. We randomly assigned them to view 1 of the 2 videos. After viewing, we first asked the participants to rate the perceived gamification and then asked them to rate the perceived enjoyment (α=.76) and favorability (α=.87) toward the Douyin CRM program. Confounding variables (ie, brand trust, brand attitude, and brand familiarity), as in Study 1, were also checked, and they showed no significant differences between the 2 groups (*P*=.08, *P*=.14, *P*=.12, respectively). All the measures were the same as those in Study 1.

We performed the manipulation check on the variable of perceived gamification in Study 2, replicating the results of the pretest. The 2 videos were significantly different in levels (lower gamification mean 4.36; higher gamification mean 5.77; *F*_1,112_=33.87, *P<*.001).

As in Study 1, we conducted a series of analyses of variance (ANOVAs) on the dependent variables. The results showed that gamification positively influenced enjoyment (lower gamification mean 5.21; higher gamification mean 5.94; *F*_1,112_=19.57, *P<*.001; partial ƞ^2^=0.15), with higher gamification leading to more enjoyment. The results also revealed that gamification had a positive impact on favorability (lower gamification mean 5.67; higher gamification mean 6.05; *F*_1,112_=11.18, *P=*.001; partial ƞ^2^=0.09), with higher gamification leading to higher favorability.

**Figure 2:**
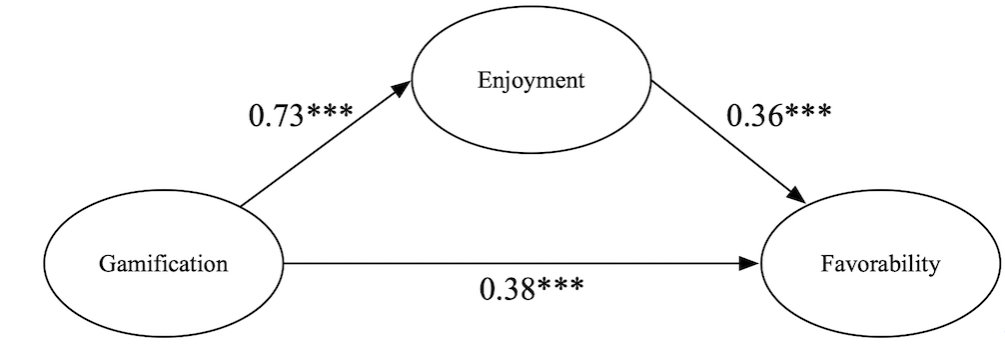
Path coefficients of the mediation model. ***P<.001.

To test H2, we conducted a mediation analysis using the bootstrap method (5000 bootstrap samples) in the mediation package of R. The results indicated that the effect of gamification on favorability was fully mediated by enjoyment. As Figure 2 illustrates, the total effect of gamification on favorability was significant (β=.38, *P<*.001). Controlling for gamification, enjoyment also had a significant effect on favorability (β=.36, *P<*.001). However, controlling for enjoyment, gamification no longer had a significant influence on favorability (β=.12, *P*=.17. The indirect effect of gamification on favorability through enjoyment was 0.730.36=0.26 (*P<*.001), with the 95% CI ranging from 0.16 to 0.63, excluding 0. Thus, H2 was supported.

Study 2 not only reinforced H1 but also gave support to our hypothesis on the mediation role of enjoyment (H2) between gamification and favorability.

### Results of Study 3

#### Pretest

We performed a pretest with 30 participants recruited from Credamo. Participants were randomly assigned to 1 of 2 conditions, and there were no significant differences between the 2 groups in brand trust (lower gamification mean 5.66; higher gamification mean 5.48; *P*=.58), brand attitude (lower gamification mean 5.55, higher gamification mean 5.73; *P*=.66), or brand familiarity (lower gamification mean 6.14; higher gamification mean 6.06; *P*=.84). However, perceived gamification differed between the 2 conditions (lower gamification mean 5.21; higher gamification mean 6.12; *F*_1,28_=8.62, *P=*.007).

#### Main Study

Study 3 used a 2 (gamification: lower, higher) 2 (rules presentation: without visual cues, with visual cues) between-subjects design. We recruited a new sample of 250 participants from Credamo; 128 (51.2%) were female, and all the participants were aged from 21 years to 40 years. In the videos, we first presented the rules (with or without visual cues) for “Protect Pandas” and then showed the interfaces of the program. After viewing, the participants were asked to rate the perceived gamification, enjoyment, and favorability toward the CRM program as in the previous studies. A confounding check was conducted and revealed no significant differences (*P=*.30, *P*=.78, *P*=.70, respectively).

Similar to the results of the pretest, the results of the manipulation check on the variable of perceived gamification showed that the 2 videos were significantly different in levels (lower gamification mean 5.16; higher gamification mean 5.72; *F*_1,248_=19.48, *P<*.001).

**Figure 3:**
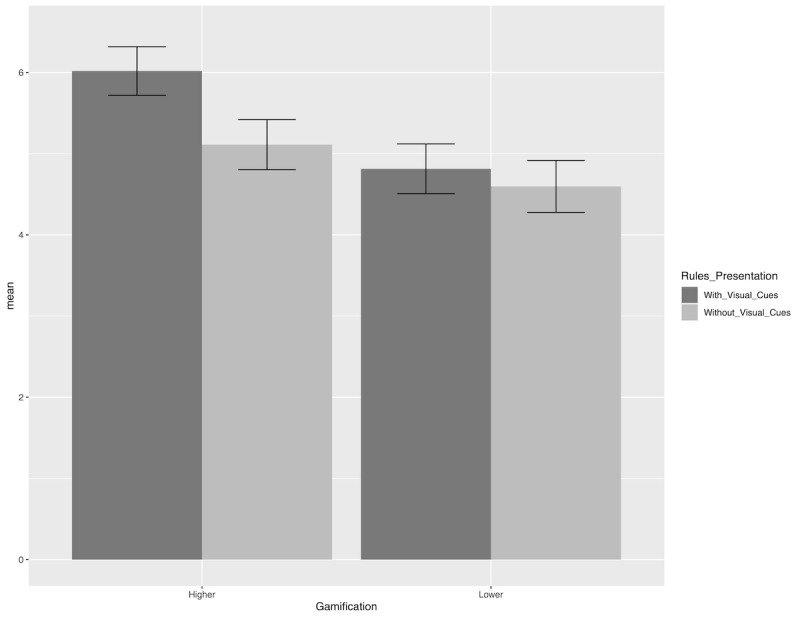
Mean (SD) enjoyment in the higher gamification group compared with the lower gamification group.

We first conducted an ANOVA with enjoyment as the dependent variable and gamification and rules presentation as the independent variables. The results confirmed the main effect of gamification on enjoyment (lower gamification mean 4.71; higher gamification mean 5.56; *F*_1,246_=30.06, *P<*.001; partial ƞ^2^=0.10) and the main effect of rules presentation (without visual cues mean 4.86; with visual cues mean 5.43; *F*_1,246_=13.91, *P<*.001; partial ƞ^2^=0.05). These simple effects were also qualified by a significant gamification rules presentation interaction effect on enjoyment (*F*_1,246_=4.98, *P*=.03; partial ƞ^2^=0.02). The planned contrast revealed that, when rules were presented with visual cues, higher gamification led to more enjoyment than did lower gamification (lower gamification mean 4.81; higher gamification mean 6.02; *F*_1,246_=30.92, *P<*.001). Meanwhile, when rules were presented without visual cues, there was also a significant difference between the 2 groups (lower gamification mean 4.60; higher gamification mean 5.11; *F*_1,246_=5.43, *P*=.02). See Figure 3.

**Figure 4:**
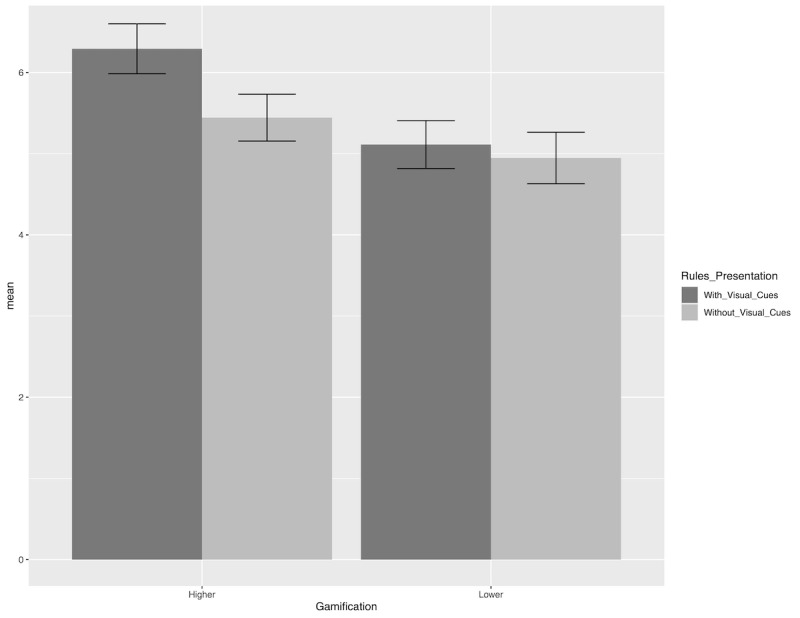
Mean (SD) favorability in the higher gamification group compared with the lower gamification group.

We next performed an ANOVA with favorability as the dependent variable and gamification and rules presentation as the independent variables. The results confirmed the main effect of gamification on favorability (lower gamification mean 5.03; higher gamification mean 5.86; *F*_1,246_=29.93, *P<*.001; partial ƞ^2^=0.10) and the main effect of rules presentation (without visual cues mean 5.21; with visual cues mean 5.72; *F*_1,246_=11.83, *P<*.001; partial ƞ^2^=0.04). These simple effects were also qualified by a significant gamification rules presentation interaction effect on favorability (*F*_1,246_=5.08, *P*=.03; partial ƞ^2^=0.02). The planned contrast revealed that, when rules were presented with visual cues, higher gamification led to more favorable attitudes toward the CRM program than did lower gamification (lower gamification mean 5.11; higher gamification mean 6.29; *F*_1,246_=30.92, *P<*.001). Meanwhile, when rules were presented without visual cues, there was also a significant difference between the 2 groups (lower gamification mean 4.95; higher gamification mean 5.44; *F*_1,246_=5.29, *P*=.02). See Figure 4.

Finally, we estimated our moderated mediation model (in the mediation package of R; 5000 bootstrap samples) to test whether the visual cues in rules representation moderate the underlying process via enjoyment. The model used gamification as the independent variable, enjoyment as the mediator, and visual cues as the moderator. Similar to Study 1 and Study 2, gamification positively influenced enjoyment (*β*=.51, *t*_246_=2.33, *P*=.02). Moreover, the interaction between gamification and visual cues was significant for enjoyment (*β*=.69, *t*_246_=2.23, *P<*.03). Enjoyment, in turn, facilitated favorability (*β*=.87, *t*_245_=30.80, *P<*.001). Visual cues significantly moderated the indirect effect of gamification on favorability via enjoyment (index of moderated mediation: 95% CI –1.12 to –0.10; for rules presentation with visual cues: 95% CI=0.69 to 1.40; for rules presentation without visual cues: 95% CI 0.08 to 0.83). However, the direct path from gamification to favorability was not significant (*P*=.61) nor was its moderation by visual cues (95% CI –0.39 to 0.20).

In summary, the findings from Study 3 supported our hypotheses (H1 to H3). Gamified CRM projects can induce more enjoyment and higher favorability. Importantly, when rules were presented with visual cues, the impact of gamification on enjoyment was more positive than when without visual cues. The results highlight the notion that consumers perceive enjoyment from not only game design elements of the CRM program but also the rules presentation.

## Discussion

﻿Across 3 studies, we showed that gamified CRM programs can increase consumers’ perceptions of enjoyment and, in turn, enhance their favorability toward the program (Studies 1 and 2). We also showed that the relationship between gamification and enjoyment is moderated by the visual cues of rule presentation (Study 3). ﻿Our findings have several implications for marketing research and practice.

Theoretically, our research contributes to the CRM literature by highlighting the impact of gamification. It is not always easy to get consumers’ responses to or willingness to participate in a CRM program (eg, Chuah et al [6] and Jun et al [48]). However, in this article, we showed that gamification is an effective alternative for CRM projects to gain more favorability by improving consumers' enjoyment. Moreover, our work adds to the literature exploring the impact of visual design on consumers’ psychological mechanisms. The results of our study indicate that, when rules are presented with visual cues, consumers are more likely to perceive higher enjoyment and, in turn, feel greater favorability.

Additionally, the current research yields some implementable managerial implications. On one hand, managers can gamify their CRM programs to enhance consumers’ enjoyment and favorability. For example, practitioners embed not only game design elements but also games into their projects. On the other hand, some gamified CRM programs merely present rules in monotonous words (eg, Dream Fairy in Study 2); however, according to our research, practitioners are better to present their rules with visual cues (even in a dynamic way) to induce more enjoyment.

We provide a range of evidence for our model, but there are still some limitations for future research. First, we only discussed the psychological mechanism of enjoyment between gamified CRM programs and favorability. However, there may exist multiple mediators. For example, engagement is another mediator to investigate. Since gamified CRM programs are possibly more interesting than ordinary CRM programs and provide various game design elements with which to interact, consumers may be more easily engaged [49,50]. When highly engaged, consumers are more inclined to believe the cause is connected with their lives [51] and thus are more willing to favor it [52]. Furthermore, engagement may increase the perception of one's personal role in contributing to the cause [18], which evokes an intention to participate. Second, in this article, we performed studies online and merely introduced CRM programs via videos. However, for more strict control and data collection, future studies can choose the context of a laboratory and provide opportunities for participants to conduct operations on certain programs. Finally, as the experience with gamification has not been defined clearly, the current manipulation check is limited. Future studies can develop scales for the gamification experience.

## Appendix

Multimedia Appendix 1 Table measurement items.

## Abbreviations

ANOVA: analysis of variance
CHERRIES: Checklist for Reporting Results of Internet E-Surveys
CRM: cause-related marketing
