# Supplementary material for: Impact of Gamification on Consumers’ Favorability in Cause-Related Marketing Programs: Between-Subjects Experiments
Source: JMIR Serious Games. 2023 Jan 10;11:e35756. doi: 10.2196/35756 (PMC9947917; doi:10.2196/35756)
Supplement: Multimedia Appendix 1 [file games_v11i1e35756_app1.docx]

Table measurement items

| Construct | Items | Source |
| --- | --- | --- |
| **Brand trust (1=“strongly disagree,” 7=“strongly agree”)** | | |
|  | I trust this brand. | So et al [43] |
|  | I rely on this brand. |  |
|  | This is an honest brand. |  |
|  | This brand is safe. |  |
| **Brand attitude** | | |
|  | This brand is bad/good (1=“bad,” 7=“good”) | Cian et al [44] |
|  | I dislike/like the brand (1=“dislike,” 7=“like”) |  |
|  | This brand is unpleasant/pleasant (1=“unpleasant,” 7=“pleasant”) |  |
| **Brand attitude (1=“totally unfamiliar,” 7=“very familiar”)** | | |
|  | How familiar are you with this brand? | Hyland and Birrell [45] |
